# Radiation-induced exosomal miR-21 enhances tumor proliferation and invasiveness in breast cancer: implications for poor prognosis in radiotherapy patients

**DOI:** 10.1186/s40164-024-00585-5

**Published:** 2024-12-18

**Authors:** Kyungmin Kim, Kyung Oh Jung, Sera Oh, Young-Hwa Kim, Seok-Yong Lee, Seongje Hong, Su Han Cho, Hyejin Kim, Siyeon Rhee, Gi Jeong Cheon, Keon Wook Kang, June-Key Chung, Hyewon Youn

**Affiliations:** 1 Department of Nuclear Medicine, Seoul National University College of Medicine, Seoul, Korea; 2 Biomedical Sciences, Seoul National University College of Medicine, Seoul, Korea; 3 Laboratory of Molecular Imaging and Therapy, Cancer Research Institute, Seoul National University College of Medicine, Seoul, Korea; 4 Department of Anatomy, College of Medicine, Chung-Ang University, Seoul, Korea; 5 Cancer Imaging Center, Seoul National University Hospital, #207-4, 103 Daehak-ro, Jongno-gu, Seoul, 110-799 Korea; 6 Department of Biology, Kyung Hee University, Seoul, Korea; 7 Woodang Network, Kangwondaehak-gil, Gangwon-do Korea; 8 K-BioX, Palo Alto, CA USA; 9 Stanford Cardiovascular Institute, Stanford University School of Medicine, Stanford University, Stanford, CA USA; 10 Department of Pharmacy, School of Pharmacy, Jeonbuk National University, Jeonju, Korea

**Keywords:** Radiotherapy, Radiation-induced exosomes, Exosomal miR-21, Poor prognosis of breast cancer patients

## Abstract

Radiotherapy is widely used as an effective non-surgical strategy to control malignant tumors. However, recurrence is one of common causes of treatment failure even after the effective radiotherapy. In this study, we focused on the effects of radiation-induced exosomal miR-21 on the tumor microenvironment to investigate the causes of recurrence. Analysis of the TCGA database revealed that breast cancer patients with high levels of miR-21 have significantly reduced overall survival when treated with radiotherapy compared to those who did not receive radiotherapy, indicating a high hazard ratio for miR-21 in patients undergoing this treatment. Additionally, exosomal miR-21 is found to be highly expressed in the serum of breast adenocarcinoma patients. To explore how miR-21 induces poor prognosis in irradiated breast cancer, we irradiated 4T1 cell line with low or high doses of radiation, and examined the impact of secreted exosomal miR-21 on breast cancer cell and tumor microenvironment. After 10 Gy irradiation, 4T1 cells secreted 2.20 ± 0.10 times more exosomes and exhibited a 1.85 ± 0.01-fold increase in exosomal miR-21 levels. Treatment with exosomes from 10 Gy-irradiated cancer cells led to enhanced tumor cell proliferation, wound healing, and migration. The survival rate of 10 Gy-irradiated tumor cells incubated with 10 Gy-derived exosomes increased by 2.83-fold. Moreover, the growth of subcutaneous tumors treated with 10 Gy exosomes (*n* = 13) was significantly faster compared to tumors treated with 0 Gy exosomes (*n* = 10, *P* < 0.05). In summary, our study revealed high-dose irradiation-induced exosomes were found to enhance tumor proliferation and invasiveness via the transfer of exosomal miR-21. Based on these findings, we suggest that radiation-induced exosomal miR-21 may contribute to a poorer prognosis of breast cancer patients undergoing radiotherapy.

**Supplementary Information:**

The online version contains supplementary material available at 10.1186/s40164-024-00585-5.

Dear editor,

Radiation therapy is widely used to treat breast cancer and is also an effective way to remove remaining breast cancer cells after surgical resection (mastectomy) to minimize the probability of cancer recurrence, especially in breast tissue or surrounding lymph nodes [1–3]. However, it has been reported that 3–15% of breast cancer patients experience recurrence within 10 years after treatment, and the prognosis in this case is relatively poor [4, 5]. Therefore, many studies have investigated and proposed tissue-associated or blood-associated biomarkers for predicting therapeutic responses or suggested therapeutic targets for improving prognosis after breast cancer treatment [6]. MiR-21, an onco-miR, has been clearly demonstrated to have higher expression in individuals with breast cancer [7–10]. However, the relationship between radiotherapy and miR-21 expression, as well as the impact of plasma miR-21 levels on the prognosis of breast cancer patients following radiotherapy, remains insufficiently explored.

In this study, we analyzed the relationship between radiation exposure and miR-21 levels in breast cancer patients using the TCGA database. Patients were categorized based on their radiotherapy status to assess potential differences in overall survival rates associated with miR-21 levels. Among patients who received radiotherapy (RT), those with high miR-21 levels had significantly poorer survival outcomes (*p* = 0.02, determined by log-rank test), while the overall survival rates of patients who did not receive RT were not affected by miR-21 expression (Fig. 1A). Additionally, a comparison of hazard ratios between the two groups-patients who received RT and those who did not-showed a significant difference, with high miR-21 expression correlating negatively with overall survival in the radiotherapy group (Fig. 1B-D). For experimental validation of the correlation between radiation intensity and miR-21 expression, we first evaluated the survival rate and viability of 4T1 mouse breast cancer cells under various radiation intensities. Employing the clonogenic assay, a well-established method for evaluating the survival rate of irradiated cancer cells, the number of colonies was counted 7–10 days post-irradiation. Exposure to 10 Gy radiation eliminated all cancer cells (Fig. 1E, F). Cell viability significantly decreased in a time-dependent manner up to 48 h after 10 Gy exposure (Fig. 1G). Irradiated 4T1 cells showed increased miR-21 expression, with levels rising by 1.55 ± 0.05 times at 5 Gy and 1.85 ± 0.01 times at 10 Gy compared to controls after 8 h, indicating that 10 Gy maintains elevated miR-21 levels longer (Fig. 1H). Using the EVmiRNA database, we also confirmed that exosomal miR-21 expression is upregulated in breast adenocarcinoma patients compared to healthy controls (Fig. 1I). Moreover, hsa-miR-21-5p expression was significantly higher in breast adenocarcinoma patients (Empirical *p* < 0.001) compared to other exosomal miRNAs, indicating that miR-21 is one of the most differentially expressed miRNAs in breast adenocarcinoma (Fig. 1J). For in vitro experiments, radiation intensities affect the exosome secretion. Exosome secretion increased significantly, with 5 Gy and 10 Gy resulting in 1.62 ± 0.15 and 2.20 ± 0.10-fold increases, respectively (Fig. 1K-M). The isolated exosomes were confirmed to express the exosomal marker proteins, CD63 and Alix, while lacking the cell marker protein, Calnexin. Their size was approximately 100 nm as determined by TEM and Nanosight analysis (Supplementary Fig. 1A). Additionally, exosomal miR-21 increased by 1.60 ± 0.29-fold after 5 Gy and 2.82 ± 0.28-fold after 10 Gy (Fig. 1N). We validated the transfer of exosomal miR-21 using a miR-21-Luciferase reporter system (Fig. 2A, B) and confirmed the uptake of exosomes labeled with Alexa 488-NHS (Fig. 2C). Bioluminescent signals in 4T1/miR-21-Luc2 cells were reduced by 78.28 ± 6.57% when treated with 10 Gy exosomes (Fig. 2D, E). To determine whether transfer of miR-21 could affect tumor proliferation and migration, 4T1 cells were incubated with 0, 5, and 10 Gy exosomes for 48 h. 4T1 cells incubated with 10 Gy exosomes exhibited 387.3 ± 13.42% higher proliferation than those treated with 0 Gy exosomes (Fig. 2F). The human breast cancer cell line MDA-MB-231 exhibited similar results, showing that 10 Gy irradiation led to an increase in exosomal miR-21 levels. Additionally, exosomes from cells exposed to 10 Gy irradiation significantly enhanced tumor proliferation (Supplementary Fig. 2). The survival rate of 10 Gy irradiated 4T1 cells was 2.96 ± 0.30 times higher when treated with 10 Gy exosomes (Fig. 2G, H), and xenograft tumors treated with 10 Gy exosomes grew significantly faster than those treated with 0 Gy (Fig. 2I). Moreover, migration assays showed a 1.75 ± 0.40-fold increase in 4T1 cells treated with 10 Gy exosomes compared to control (Fig. 2J, K), and wound-healing assay revealed that at 28 h, the wound distance was 24.61 ± 7.90 nm in 4T1 cells treated with 10 Gy exosomes and 61.37 ± 7.06 nm in 4T1 cells treated with 0 Gy exosomes (Fig. 2L, M). We confirmed alterations in the expression of proteins PTEN and TIMP-3, both associated with the miR-21 pathway, in 4T1 cells treated with exosomes (Supplementary Fig. 1B-D). This indicates that exosomal miR-21 can lead to activate downstream signaling, promoting tumor malignancy. We identified a potential role of exosomal miR-21 in influencing the tumor microenvironment by inducing M2 macrophage polarization (Supplementary Fig. 3). In addition, we confirmed exosomal miR-21 on fractionated radiation and validated the effects of miR-21 on tumor proliferation (Supplementary Fig. 4).

In conclusion, we have clarified the novel aspects of our study, which focuses on the effects of radiotherapy on miR-21 expression and its exosomal release in breast cancer cell lines and clinical data. We now emphasizes how different radiation doses impact exosomal miR-21 and its role in tumor proliferation and migration, as well as its potential implications for combination therapy with radiation.

**Fig. 1:**
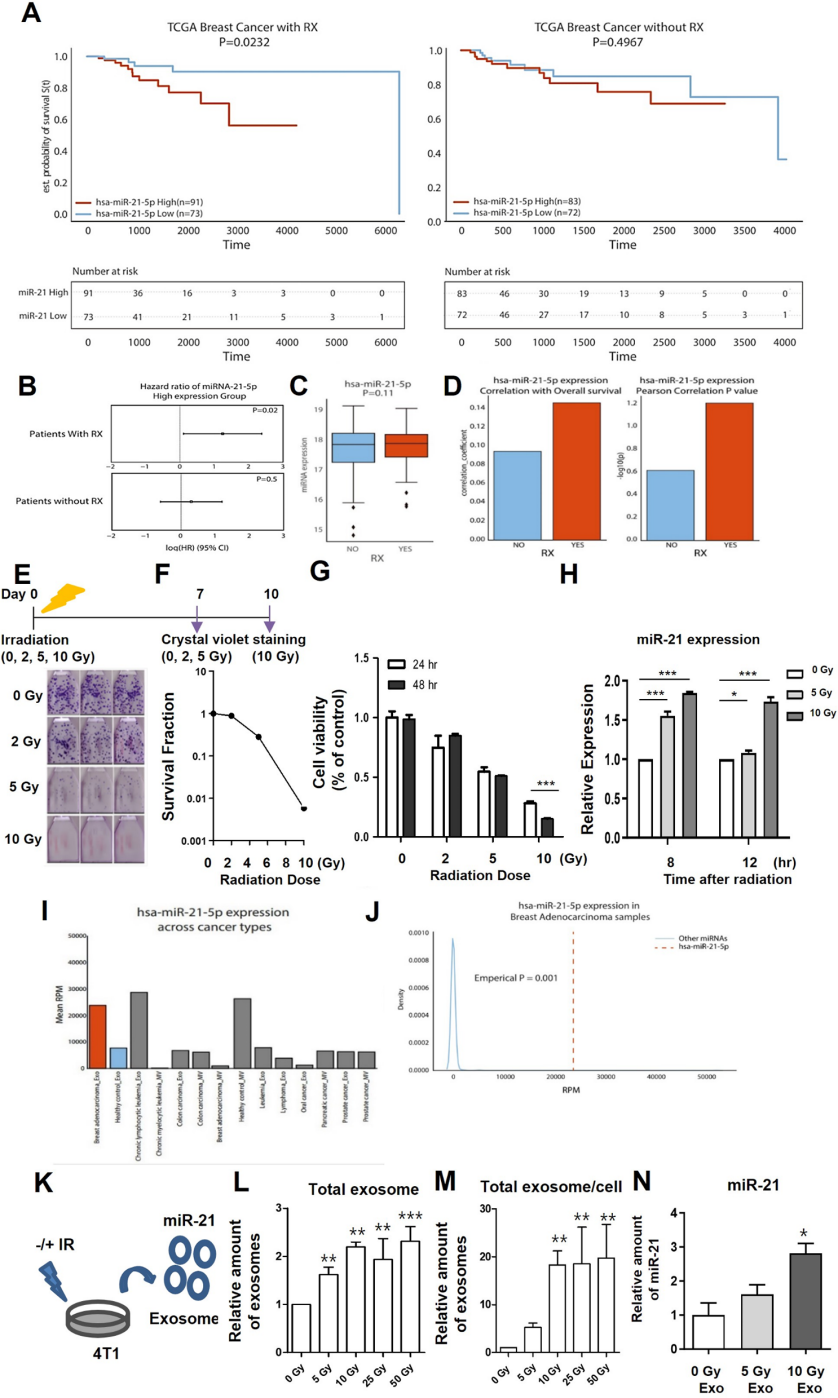
Breast cancer patients with high miR-21 levels are correlated with poor prognosis after radiation therapy. (**A**) Kaplan-Meier survival analysis of the TCGA breast cancer cohort stratified by radiotherapy status and miR-21 expression levels. Sample sizes are indicated at specific time points. (**B**) Hazard ratios for groups stratified by radiotherapy status and miR-21 expression levels. Cox proportional hazards model was used to calculate hazard ratios and corresponding p-values for each group. (**C**) Expression of has-miR-21 in breast cancer patients stratified by RX status. (**D**) Pearson correlation analysis between miR-21 expression and overall survival for each sample. (**E**,** F**) Representative images (**E**) and survival fraction (graph, **F**) of Clonogenic assay in 4T1 after irradiation (0/2/5/10 Gy). (**G**) Cell viability test in 4T1 at 24 and 48 h after irradiation (0/2/5/10 Gy). (**H**) miR-21 expression in 4T1 after irradiation (0/5/10 Gy). (**I**) Comparative analysis of exosomal miR-21 expression (RPM, reads per million) in breast adenocarcinoma and healthy control. (**J**) Distribution of exosomal miRNA expression in breast adenocarcinoma samples from the EVmiRNA database. The levels of hsa-miR-21-5p were compared with the distribution of other miRNAs available in the EVmiR database. Empirical p-value was determined based on the rank of hsa-miR-21-5p expression within the expression ranks of other miRNAs. (**K**) Experimental scheme for isolating exosomes. (**L**,** M**) The amount of secreted exosomes according to irradiation dose (0/5/10/25/50 Gy, **L**) and that normalized with viable cell number (**M**). (**N**) Quantification of exosomal miR-21 levels isolated from irradiated 4T1 (0/5/10 Gy)

**Fig. 2:**
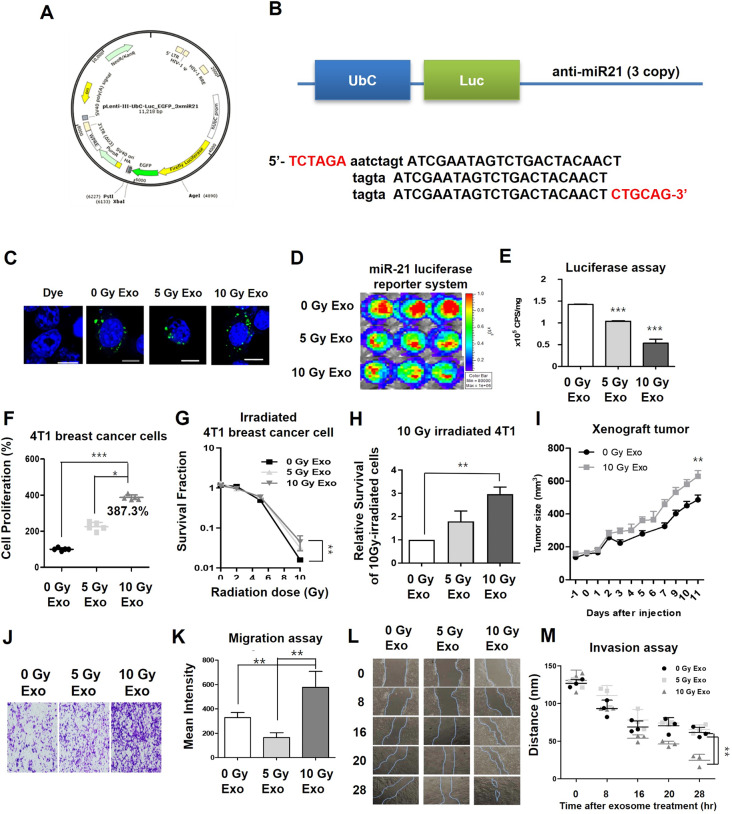
(**A**) Vector construct. (**B**) Bioluminescence imaging strategy for the miR-21 reporter system. (**C**) Fluorescence images of 4T1 with Alexa 488-labeled exosomes. (**D**, **E**) Bioluminescence images (**D**) and luciferase assay (**E**) of 4T1/miR-21-luc treated with exosomes (0/5/10 Gy). (**F**) Cell proliferation of 4T1 with exosomes (0/5/10 Gy). (**G**) Survival fraction of irradiated 4T1 with exosomes (0/5/10 Gy). (**H**) Relative survival value in 10 Gy irradiated 4T1 treated with exosomes (0/5/10 Gy). (**I**) Xenograft tumor volume with exosome (0/5/10 Gy) treatment. (**J**, **K**) Representative images of migration assay of 4T1 with exosomes (**J**) and migration ratio (graph, **K**). (**L**, **M**) Representative images of wound healing assay (**L**) and distance analysis (graph, **M**) of 4T1 with exosomes (0/5/10 Gy)

## Electronic supplementary material

Below is the link to the electronic supplementary material.

Supplementary Material 1

## Data Availability

No datasets were generated or analysed during the current study.
