# Supplementary material for: Radiation-induced exosomal miR-21 enhances tumor proliferation and invasiveness in breast cancer: implications for poor prognosis in radiotherapy patients
Source: Exp Hematol Oncol. 2024 Dec 18;13:120. doi: 10.1186/s40164-024-00585-5 (PMC11656813; doi:10.1186/s40164-024-00585-5)
Supplement: Supplementary file 1 — Supplementary Material 1 [file 40164_2024_585_MOESM1_ESM.docx]

**
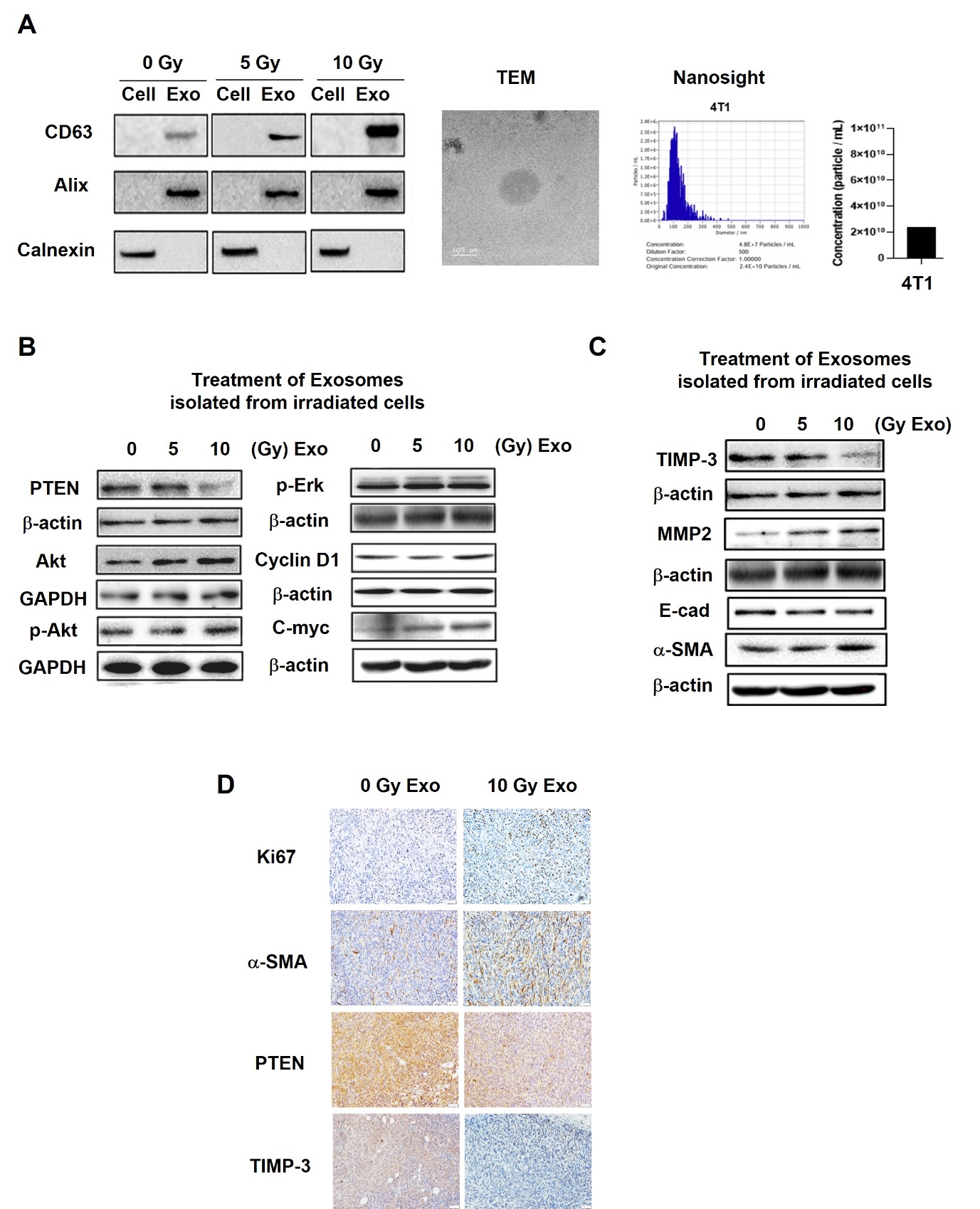
**

**Supplementary Fig. 1 Exosomal miR-21 downregulates tumor suppressor genes, activating downstream signaling pathway A** Exosome characterization by western blot for exosome marker (CD63 and Alix) and cell marker (Calnexin), along with TEM imaging and Nanosight analysis. **B** Western blot of the PTEN as a target of miR-21, and PTEN-related proteins in 4T1 breast cancer cells with exosomes (0/5/10 Gy). **C** Western blot of the TIMP-3 as a target of miR-21, and TIMP-3 related proteins in 4T1 with exosomes (0/5/10 Gy). **D** Immunohistochemistry of Ki67, a-SMA, PTEN and TIMP-3 in xenograft tumor tissues treated with 0, 10 Gy exosomes.


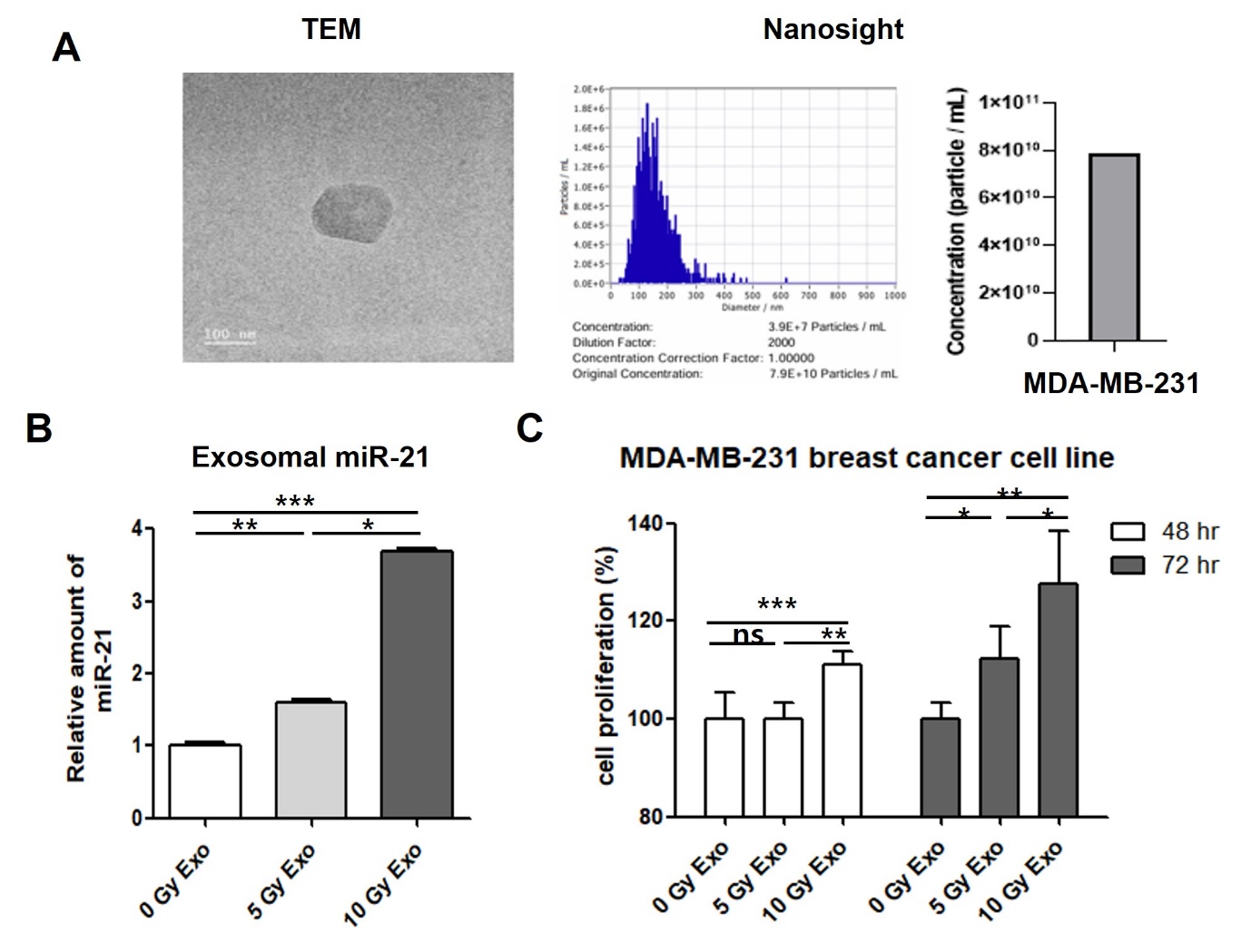


**Supplementary Fig. 2 Exosomal miR-21 levels are enhanced by high-dose irradiation in human breast cancer cell line. A** TEM image and Nanosight analysis of exosomes derived from MDA-MB-231. **B** Quantification of exosomal miR-21 levels isolated from irradiated MDA-MB-231 (0/5/10 Gy). **C** Cell proliferation of MDA-MB-231 with exosomes (0/5/10 Gy).
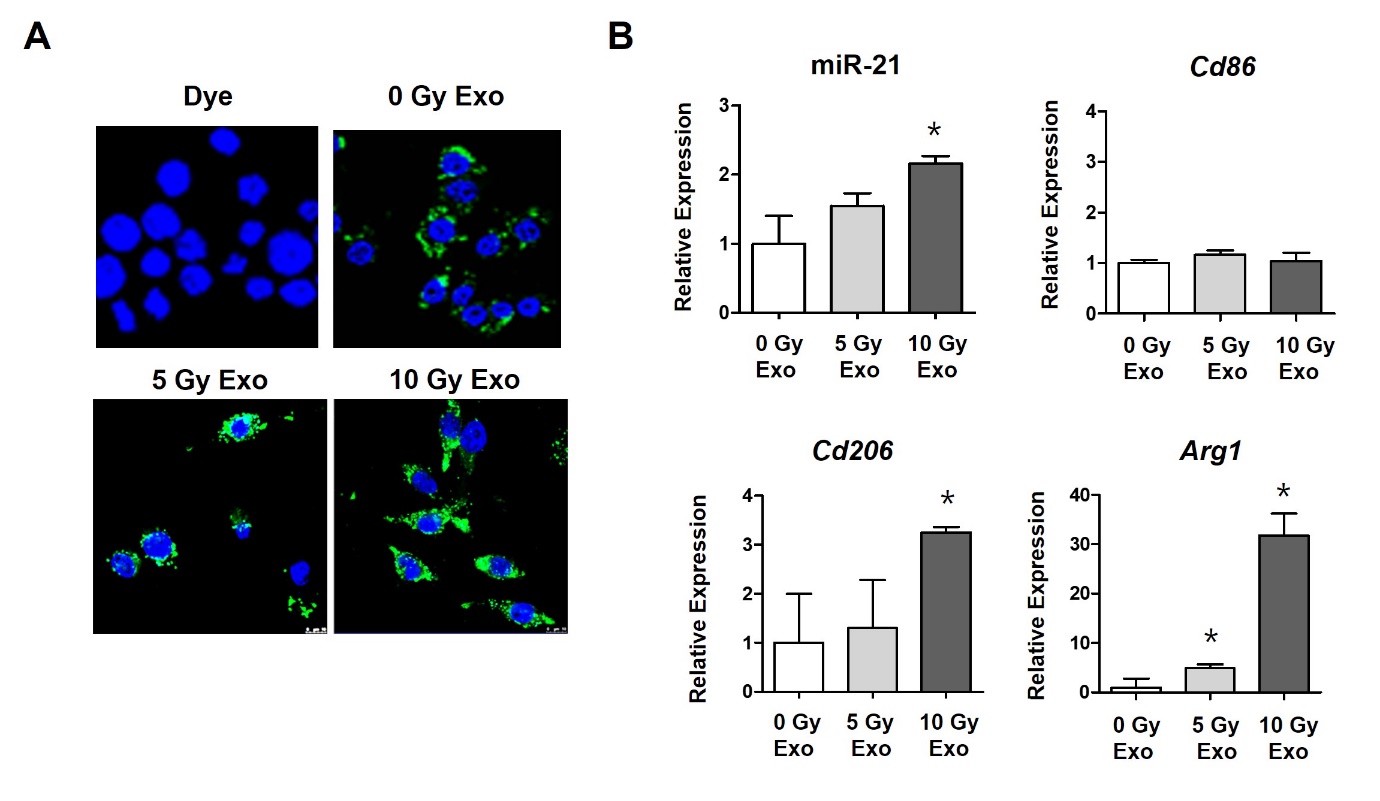


**Supplementary Fig. 3 Relevance of radiation induced exosomes and M1/M2 polarization. A** Confocal microscopy of RAW264.7 after Dye labeled Exo treatment. **B** qRT-PCR of miR-21, M1 marker (*Cd86*) and M2 marker (*Cd206* and *Arginase 1*) after exosomes treatment (0/5/10 Gy).


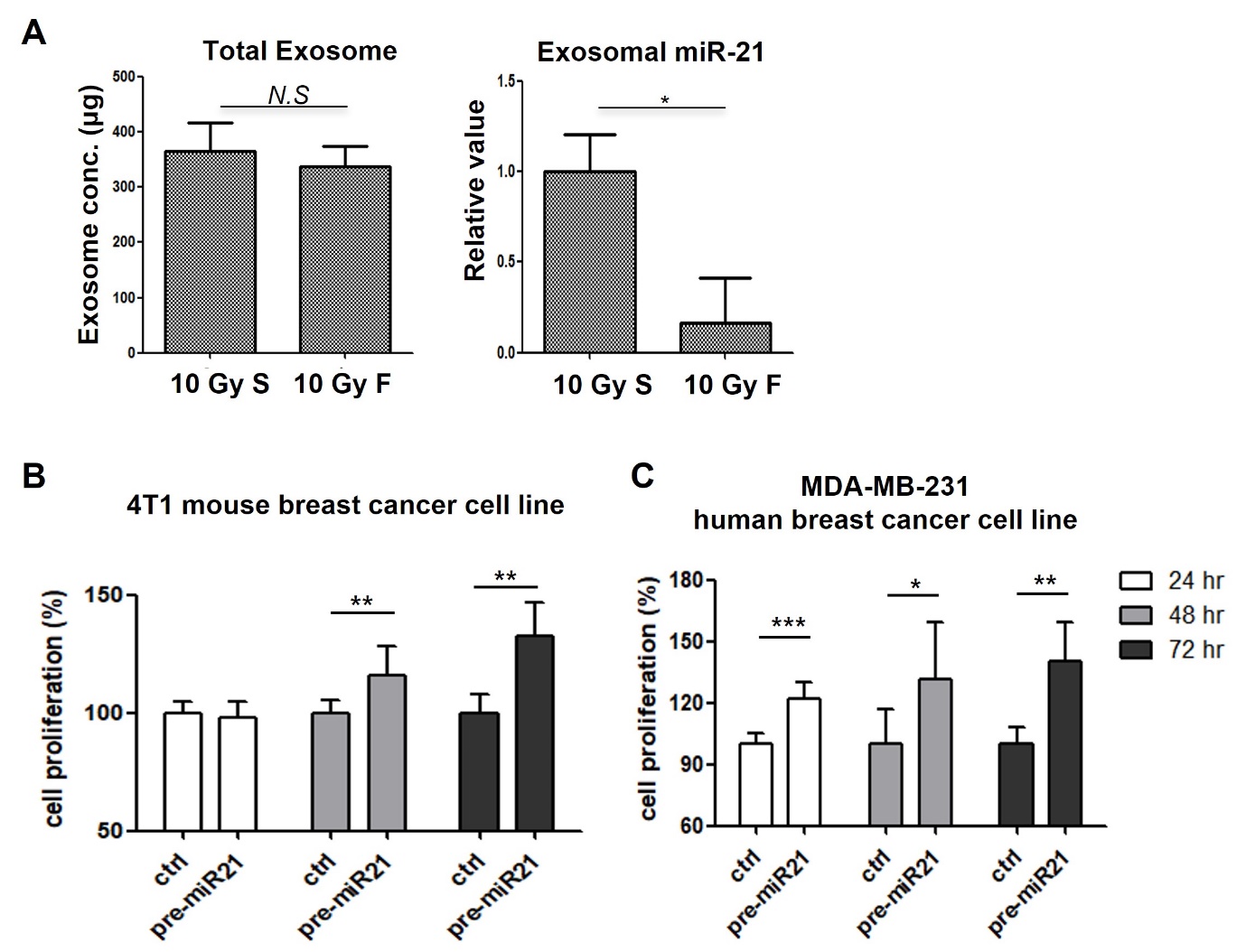


**Supplementary Fig. 4 Exosomal miR-21 was different on fractionated radiation A** Comparison of the total exosome amount and exosomal miR-21 levels between single-dose (10 Gy) and fractionated irradiation (2 Gy, 5 times). **B, C** Cell proliferation of 4T1 **(B)** and MDA-MB-231 **(C)** transfected with pre-miR-21.
